# Association mapping of important agronomic traits in *Mucuna pruriens* (L.) DC.

**DOI:** 10.1186/s40529-024-00421-3

**Published:** 2024-08-19

**Authors:** Patrush Lepcha, Mahesh Shekhar, Leelambika Murugesan, Mahammad Jaheer, Ratan Chopra, Vikas Belamkar, Narayana Sathyanarayana

**Affiliations:** 1 Department of Botany, Sikkim University, P. O, Tadong, Sikkim, Gangtok 737102 India; 2 Department of Biotechnology, Sir M, Visvesvaraya Institute of Technology, Bangalore, Karnataka 562157 India; 3 Department of Plant and Microbial Biology, University of Minnesota, Saint Paul, MN 55108 USA; 4 Department of Agronomy and Horticulture, University of Nebraska-Lincoln, Lincoln, NE 68583 USA; 5 Department of Life Science, Central University of Karnataka, Aland Road, Kadaganchi-585 367, Kalaburagi, Karnataka India

**Keywords:** Genetic diversity, Marker-trait association, Population structure, Seed traits

## Abstract

**Background:**

The tropical legume *Mucuna pruriens* (L.) DC. can meet three agricultural needs: low-cost protein, high-value medicines, and green manure or cover crops. But like other underutilized crops, it needs more modern breeding resources. Identifying marker-trait associations (MTAs) can facilitate marker-assisted breeding and crop improvement. Recent studies have demonstrated the feasibility of identifying MTAs using a small number of accessions (< 100). We have characterized a panel of 70 *M. pruriens* accessions across two consecutive years and performed association analysis for 16 phenotypic traits related to seed (seed length, seed width, seed thickness, seed yield per plant, hundred seed weight); pod (pod length, pod width, number of pods per cluster, number of pods per plant); inflorescence (inflorescence length, flower buds per inflorescence, flower length, pedicel length), and biochemical attributes (L-DOPA, total protein, total carbohydrate), using 66 genic-microsatellite markers following mixed linear model.

**Results:**

The results showed significant phenotypic (*P* < 0.05) and genetic diversity (Shannon’s information index, I = 0.62) in our germplasm collection. Many tested traits were highly heritable (broad-sense heritability ranging from 42.86 to 99.93%). A total of 15 MTAs was detected at an adjusted significance level of *P* < 5.55 × 10^− 3^ for nine traits (seed length, seed thickness, seed width, hundred seed weight, seed yield per plant, inflorescence length, flower buds per inflorescence, flower length, and petiole length), contributed by 10 SSR markers (MPU_19, MPU_42, MPU_54, MPU_57, MPU_58, MPU_83, MPU_89, MPU_108, MPU_111, and MPU_122.) with phenotypic variance explained (PVE) ranging from 14.7 to 31.1%. Out of the ten trait-associated markers, the BLAST analysis revealed putative functions of seven markers, except MPU_57, MPU_58, and MPU_83.

**Conclusion:**

Fifteen MTAs identified for important traits with phenotypic variance explained > 10% from mixed linear model offer a solid resource base for improving this crop. This is the first report on association mapping in *M. pruriens* and our results are expected to assist with marker-assisted breeding and identifying candidate genes in this promising legume.

**Supplementary Information:**

The online version contains supplementary material available at 10.1186/s40529-024-00421-3.

## Background

Tapping the potential of neglected and underutilized species is vital to reduce overreliance on staples and improve global food security. The prospects of *Mucuna pruriens* (L.) DC. for tropical agriculture is well recognized (Pugalenthi et al. 2005). Its important traits include edible protein and the production of L-DOPA (l-3,4-dihydroxyphenylalanine), used in treating Parkinson’s disease. Identification of marker-trait association can accelerate marker-assisted breeding programs and propel its use for commercial cultivation.

*M. pruriens* is a member of the phaseloid clade of Leguminosae/Fabaceae. It is a self-pollinated annual species with a diploid genome (2n = 2x = 22) of approximately 1281 to 1361 Mbp (Sastrapradja et al. 1974; Sathyanarayana et al. 2017) and occurs both in the wild (var. *pruriens* and var. *hirsuta*) and cultivated (var. *utilis*) forms (Wilmot-Dear 1987; Sasidharan 2004). Earlier works reported wide-ranging taxonomic confusions within this species (Duke 1981). The taxonomic revision carried out by Wilmot Dear (1987) showed that many of the previously reported species (*M. aterrima, M. cochinchinensis, M. hassjoo, M. nivea, and M. utilis*) as mere varieties of *M. pruriens* suggesting their inclusion in one of three botanical varieties [viz., var. *pruriens* and var. *hirsuta* (wild forms) and var. *utilis* (cultivated forms)]. Further examination of these varieties based on morphometric and RAPD markers found close genetic similarities and breeding compatibility between var. *hirsuta* and var. *pruriens*, proposing their merger into a single botanical variety (var. *pruriens*) (Leelambika and Sathyanarayana 2011). However, clear affirmation on this from combined classical and modern taxonomic tool is awaited, and until then, var. *hirsuta* conceivably needs to be maintained separately to keep uniformity in literature. Regarding agronomic benefits, with a seed-to-seed duration of 90–120 days, *M. pruriens* flourishes well under acidic soil (pH < 5.8), elevation below 1600 m, annual rainfall > 400 mm, warm (19–27 °C) and moist tropical climatic conditions (Pugalenthi et al. 2005; Kumar and Saha 2013). The plant is native to Eastern India and Southern China (Duke 1981; Wilmot-Dear 1987) but is now distributed across Asia, the Americas, the West Indies, Africa, and the Pacific Islands (Fung et al. 2011). In Southeast Asia, it is mainly found in India, Nepal, Bangladesh, Myanmar, Sri Lanka, and Malaysia (Fung et al. 2011; Kumar and Saha 2013). Within the Indian sub-continent, its distribution ranges from the lower Himalayan range to the entire tropical plains of India (Muralia and Pathak 2003). It is adapted to less fertile and dry soils (Siddhuraju et al. 2000), produces a seed yield of about 1.3–2.4 t/ha (Kumwenda and Gilbert 1998; Gurumoorthi et al. 2003), and possesses disease-resistant (Eilitta et al. 2002), nematicidal (Carsky and Ndikawa 1998) as well as allelopathic properties (Fujii et al. 1991). *M. pruriens* (var. *utilis*) contains high seed protein (20–30%) akin to other edible legumes such as soybean (35–40%), chickpea (18.3–25.2%), and pigeon pea (19–21.7%) (Kumar et al. 1991; Hira and Chopra 1995; Mang et al. 2016) and thus offers a cheap source of edible protein.

Genetic diversity is a critical determinant in the success of any crop improvement program. In *M. pruriens*, earlier works found wide-ranging phenotypic and trait-specific variability in different germplasm collections (Mahesh and Sathyanarayana 2011, 2015; Sathyanarayana et al. 2012). DNA markers such as randomly amplified polymorphic DNA (RAPD) (Padmesh et al. 2006; Leelambika et al. 2010; Patil et al. 2016), inter simple sequence repeat (ISSR) (Patil et al. 2016; Chinapolaiah et al. 2018), amplified fragment length polymorphism (AFLP) (Sathyanarayana et al. 2011; Mahesh and Sathyanarayana 2015; Tripathi et al. 2018) and simple sequence repeat (SSR) (Shetty et al. 2015; Sathyanarayana et al. 2017; Kumar et al. 2019) have reinforced these findings. Regarding sequence information, the first transcriptome analysis reported *de novo* assembly, functional annotation, and differential gene expression among pod, leaf, and root tissues (Sathyanarayana et al. 2017). In another study that followed, detailed transcriptome analysis was carried out along with biochemical characterization to track the presumptive biosynthetic pathway genes associated with L-DOPA production (Singh et al. 2018). More recently, Yuan et al. (2021) have generated sequencing of the plastid genome of 152,119 bp, while a whole genome sequencing effort by Hao et al. (2022) has created 500.49 Mb sequence data across 11 chromosomes.

In molecular breeding, studies aiming at developing linkage maps and tagging agronomic traits using AFLP markers were initiated (Capo-chichi et al. 2004; Mahesh et al. 2016). However, these efforts didn’t advance beyond preliminary findings, perhaps due to resource constraints often faced by such underutilized crops. It is well known that conventional linkage analysis is both time and resource-intensive due to the requirement of bi-parental populations. It also suffers from efficacy issues due to low-resolution and limited allelic variation (Flint-Garcia et al. 2005; Yu and Buckler 2006; Oraguzie and Wilcox 2007; Abdurakhmonov and Abdurakhmov 2008). Alternatively, association mapping offers a powerful method for identifying marker-trait association as it determines the relationship between phenotypic variations and genetic polymorphisms considering naturally occurring or historical recombination (Ambreen et al. 2018). It has been successfully used for identifying marker-trait associations (MTAs) in different legume species (Liu et al. 2017; Singh et al. 2017; Zhao et al. 2017; Vaijayanthi et al. 2018). However, no study has explored this opportunity in *M. pruriens*. Among the molecular markers, EST-SSRs have been proven reliable for association analysis as they are directly linked with the genes expressing a trait (Varshney et al. 2005). Several species-specific EST-SSR markers have recently been developed using RNA-seq analysis in *M. pruriens* (Sathyanarayana et al. 2017). Plant breeders also often choose the SSR markers due to their co-dominant nature, multiallelic expression, high polymorphism, and greater genome abundance (Kalia et al. 2011).

For association mapping, core collections have often been effective, but core collections of adequate size are generally available only for selected/mainstream crop species (Liu et al. 2017; Zhao et al. 2017; Ambreem et al. 2018). For crops that lack a large and established core collection, a diverse panel of accessions from the germplasm collection can be used, provided they demonstrate the presence of high genetic variance, low kinship association among its individuals, and lack of strong population structure (Hu et al. 2014; Mahajan et al. 2017). Association analysis is usually carried out on a large number of accessions (> 300). But in cases where the numbers are limited, it is still possible to explore MTAs (Soumya et al. 2021). Recently, several genome-wide association studies (GWAS) have been successfully carried out in population sizes between 60 and 150 (Lehnert et al. 2018; Ma et al. 2018; Rohilla et al. 2020). Taking cues from these studies, in the present work, we evaluated a panel of seventy diverse *M. pruriens* accessions for suitability as an association mapping panel and performed association analysis on sixteen agronomically important traits using microsatellite markers.

## Materials and methods

### Plant material and phenotypic evaluation

As an association mapping panel, we used a subset of 70 *M. pruriens* accessions from our germplasm collection. This comprised 11 accessions of *M. pruriens* var. *utilis* (cultivated variety), 28 of var. *pruriens* (wild variety), and 31 of var. *hirsuta* (wild variety). They represented three large geographic regions in India - Eastern, Southern, and West-Central India (Table S1). Sixteen traits related to seed (seed length, seed width, seed thickness, seed yield per plant, hundred seed weight); pod (pod length, pod width, number of pods per cluster, number of pods per plant); inflorescence (inflorescence length, flower buds per inflorescence, flower length, pedicel length), and biochemical attributes (L-DOPA, total protein, total carbohydrate) were evaluated for two consecutive years (2014 and 2015). We used manual measurements using a vernier caliper, weighing balance, and counting as relevant for the trait for data scoring. The L-DOPA content was estimated as per the method of Daxenbichler et al. (1972). The total protein and carbohydrate contents were estimated using Lowry’s (Lowry et al. 1951) and Anthrone’s (Hedge and Hofreiter 1962) methods, respectively. All statistical analyses, such as one-way analysis of variance (ANOVA), correlation, and principal component analysis (PCA), were performed using the R program (R Core Team 2014).

### Estimation of variance components and broad-sense heritability

To estimate the variance, genotypic and phenotypic coefficients of variation were evaluated using Syukur et al. (2012) as follows:

σ^2^G = (MSG – MSE) / r.

σ^2^P = σ^2^G + σ^2^E/r.

Where, σ^2^G = genotypic variance, σ^2^P = phenotypic variance, σ^2^E = environmental variance (error mean square from the analysis of variance); MSG = mean square of genotypes; MSE = error mean square; r = number of replications.

The broad-sense heritability for each trait was estimated according to Allard (1960) as follows:

H^2^ = (σ^2^G/ σ^2^ P) × 100.

Where, H^2^ = heritability in broad-sense; σ^2^G = genotypic variance; σ^2^P = phenotypic variance.

### DNA isolation and SSR genotyping

Leaf tissues from 15-day-old seedlings were used for the DNA isolation as described previously (Doyle and Doyle 1990). Genotyping was done using a sub-set of 90 species-specific genic SSR primer pairs (Table S2) chosen from Sathyanarayana et al. (2017). Polymerase chain reaction (PCR) mixture included template DNA (50ng/µl), primers (1µM each), dNTPs (2.5mM), Taq polymerase (1U), PCR buffer (1X), and MgCl_2_ (1.5mM), maintaining the final volume of 25 µl. DNA amplification condition comprised of initial denaturation at 94 °C for 3 min followed by 35 cycles of 94^°^C for 30s, appropriate annealing temperature (*Tm*) for 30 s, and an extension at 72 °C for 20 s with a final extension of 7 min at 72 °C. Subsequently, one (1) µl each of PCR amplicons generated with different dye-labeled primers were mixed with 2.95 µl distilled water, 7 µl of formamide and 0.05 µl of the GeneScan™ 500 LIZ® Size Standard. Denatured DNA was size fractioned with capillary electrophoresis. As each SSR primer-pair produced multiple amplicons, primer-pairs are referred to as SSR markers and, amplicons as bands or alleles from here onwards. We applied stringent filtering criteria (minor allele frequency > 0.05 and missing percentage less than 20%) for the marker bands produced. Those SSRs fulfilling these criteria were retained and used for further analysis.

### Marker attributes and genetic diversity

Marker attributes such as polymorphism information content (PIC) and major allele frequency (MAF) were calculated by PowerMarker v 3.25 (Liu and Muse 2005). The total number of alleles (Na), effective number of alleles (Ne), observed heterozygosity (Ho), expected heterozygosity (He), percentage of polymorphic loci (%P), gene flow (Nm), and Hardy-Weinberg equilibrium (HWE) were assessed by GenAlEx v 6.5 (Peakall and Smouse 2012). HP-Rare v 1.0 (Kalinowski 2005) was used to determine private/rare allelic richness per locus (R_p_) based on rarefaction approach. The genetic diversity indices such as - Shannon’s information index (I), Nei’s gene diversity (h), and total genetic diversity (H_T_) were determined by using POPGENE v 1.32 (Yeh et al. 1999).

### Population structure, relative kinship, and genetic relationship

Population structure was analyzed using the Bayesian clustering method implemented in STRUCTURE v 2.3.4 (Pritchard et al. 2000). For each accession, the proportion of ancestral contribution was estimated using the admixture model and correlated allele frequencies. K-values ranging from 1 to 10 were tested with ten independent replications, 100,000 lengths of the burn-in period, and 200,000 Markov Chain Monte Carlo (MCMC) repetitions for each K. The optimal K-value was obtained using STRUCTURE analysis results with STRUCTURE HARVESTER (Earl 2012). Accessions were assigned to a subgroup depending on the Q-value (membership proportion), and if the Q-value was < 80%, they were termed admixtures. Molecular variance (AMOVA) and pairwise F_ST_ of geographic population and subpopulations were analyzed using GenAlEx v 6.5 (Peakall and Smouse 2012) with 1,000 permutations. The kinship coefficients (Fij; individual level) were calculated following Loiselle et al. (1995) to estimate the relatedness between the individuals. TASSEL v 5.0 (Bradbury et al. 2007) was used to generate the kinship coefficient matrix among all pairs of accessions. The kinship heat map was obtained using GAPIT, R package (Wang et al. 2014). Genetic relationships based on the distance were arrived at through the construction of an unrooted neighbor-joining (NJ) dendrogram and principal coordinate analysis (PCoA) using Darwin v 6.0 (Perrier and Jacquemoud-Collet 2006). The reliability of the NJ dendrogram was tested with the bootstrap value of 1000 replicates.

### Association mapping and annotation of the MTAs

TASSEL v 5.0 (Bradbury et al. 2007) was used for the association analysis. A mixed linear model (MLM) was employed to determine the MTAs. The MLM is considered superior over the general linear model (GLM), as GLM incorporates only population structure (Q matrix), which often results in false-positives; instead, MLM incorporates both Q and K matrix, which overcomes this limitation (Yu et al. 2006). Generally, in such studies, Bonferroni multiple test correction (*P* = 0.05/n; n is the number of markers used in the study) is applied to obtain the threshold *P*-value as it provides a stringent cut-off to avoid false positives. However, it assumes that the loci are independent, which is not always true, given that certain loci may be in linkage disequilibrium. Therefore, to avoid loss of beneficial MTAs, we also used a less stringent criterion, *P* = 1/n as an alternative cut-off in addition to Bonferroni correction. This approach has been beneficially employed in some earlier studies (Li et al. 2012; Yang et al. 2014; Xu et al. 2018). The Manhattan plots and quantile-quantile (Q-Q) plots for MTAs were generated using the CMplot R package (Yin et al. 2021). The putative function of each trait-associated marker was determined using the *Arabidopsis thaliana* (L.) Heynh. genome database (https://www.arabidopsis.org). For this, the transcripts of SSR markers were used as a query sequence and matched against the reference genome sequences of *A. thaliana* with the BLAST tool. Then, the top hit was selected as the putative gene/function of the respective marker. An *e-value* ≤ 1e-05 was used as a threshold.

## Results

### Phenotypic variability

**Fig. 1:**
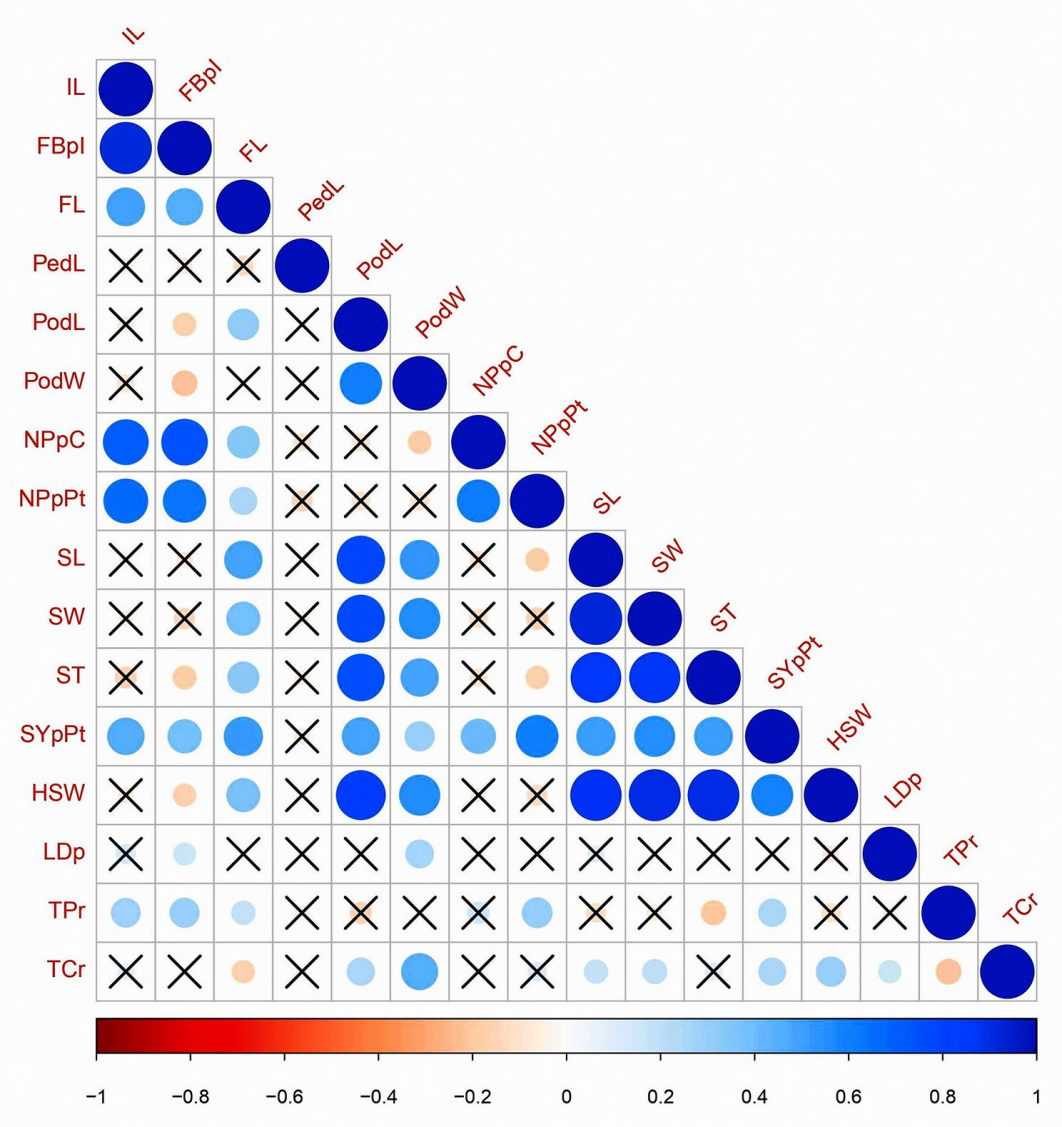
Correlation matrix of sixteen phenotypic traits in *M. pruriens* association mapping panel. Note: The intensity of color ranges from blue (positive) to brown (negative) and the size of the circles show strength of significant correlation (*P* < 0.05) IL: Inflorescence length; FBpI: Flower buds per inflorescence; FL: Flower length; PedL: Pedicel length; PodL: Pod length; PodW: Pod width; NPpC: Number of pods per cluster; NPpPt: Number of pods per plant; SL: Seed length; SW: Seed width; ST: Seed thickness; SYpPt: Seed yield per plant; HSW: Hundred seed weight; LDp: Total L-DOPA; TPr: Total protein; TCr: Total carbohydrate; **×**: Statistically non-significant correlation values

The one-way ANOVA revealed significant variability (*P* < 0.05) for most phenotypic traits evaluated in our association mapping panel, except for pedicel length, flower length, and L-DOPA content. Inflorescence length and flower length showed the highest (CV = 84.97%) and the lowest variations (CV = 6.90%) (Table 1). The values of correlation coefficients indicated a significantly high positive correlation (*R* > 0.70) between some important seed, pod, and inflorescence traits (Fig. 1). For instance, hundred seed weight was strongly correlated (*R* > 0.70) with seed thickness, seed width, seed length, and pod length, and moderately correlated (0.30 < *R* < 0.70) with pod width and flower length. A similar trend was observed for seed yield as well.

**Table 1:** Variability and broad-sense heritability of sixteen seed-based traits in *M. pruriens* association mapping panel

Traits (unit of measurement)	Range	Average ± SE	CV(%)	ANOVA	H^2^(%)
Inflorescence length (cm)	1.60–93.00	18.85 ± 2.05	84.97	**	96.93
No of flower buds per inflorescence	4.25–83.75	25.54 ± 2.40	73.37	**	62.23
Flower length (cm)	4.05–5.18	4.61 ± 0.04	6.90	ns	52.94
Pedicel length (cm)	0.30–4.50	0.60 ± 0.07	80.45	ns	42.86
Pod length (cm)	6.43–14.03	8.80 ± 0.21	18.71	***	99.93
Pod width (cm)	1.43–2.55	1.82 ± 0.03	14.07	***	98.14
No of pods per cluster	2.50–28.00	10.83 ± 0.77	55.71	*	98.83
No of pods per plant	27.25-405.75	147.43 ± 11.66	61.77	**	96.77
Seed length (mm)	7.73–16.90	10.88 ± 0.28	20.19	***	96.62
Seed width (mm)	6.15–12.77	8.18 ± 0.20	19.17	***	93.92
Seed thickness (mm)	3.01–9.61	5.33 ± 0.18	26.08	***	97.27
Seed yield per plant (gm)	41.13-1136.60	303.61 ± 30.94	79.60	***	93.34
Hundred seed weight (gm)	12.83-146.36	41.71 ± 3.75	70.31	***	95.93
L-DOPA (%)	0.95–3.24	2.07 ± 0.06	22.81	ns	62.50
Total protein (%)	15.07–29.19	22.94 ± 0.42	14.14	*	98.58
Total carbohydrate (%)	6.54–28.25	14.60 ± 0.68	36.44	**	95.71

SE: Standard error; CV: Coefficient of variation; ANOVA: One-way analysis of variance; H^2^: Broad-sense heritability

ns: Statistically not significant at *P* < 0.05

***: Statistically significant at *P* < 0.001

**: Statisticallysignificant at *P* < 0.01

*: Statisticallysignificant at *P* < 0.05

**Fig. 2:**
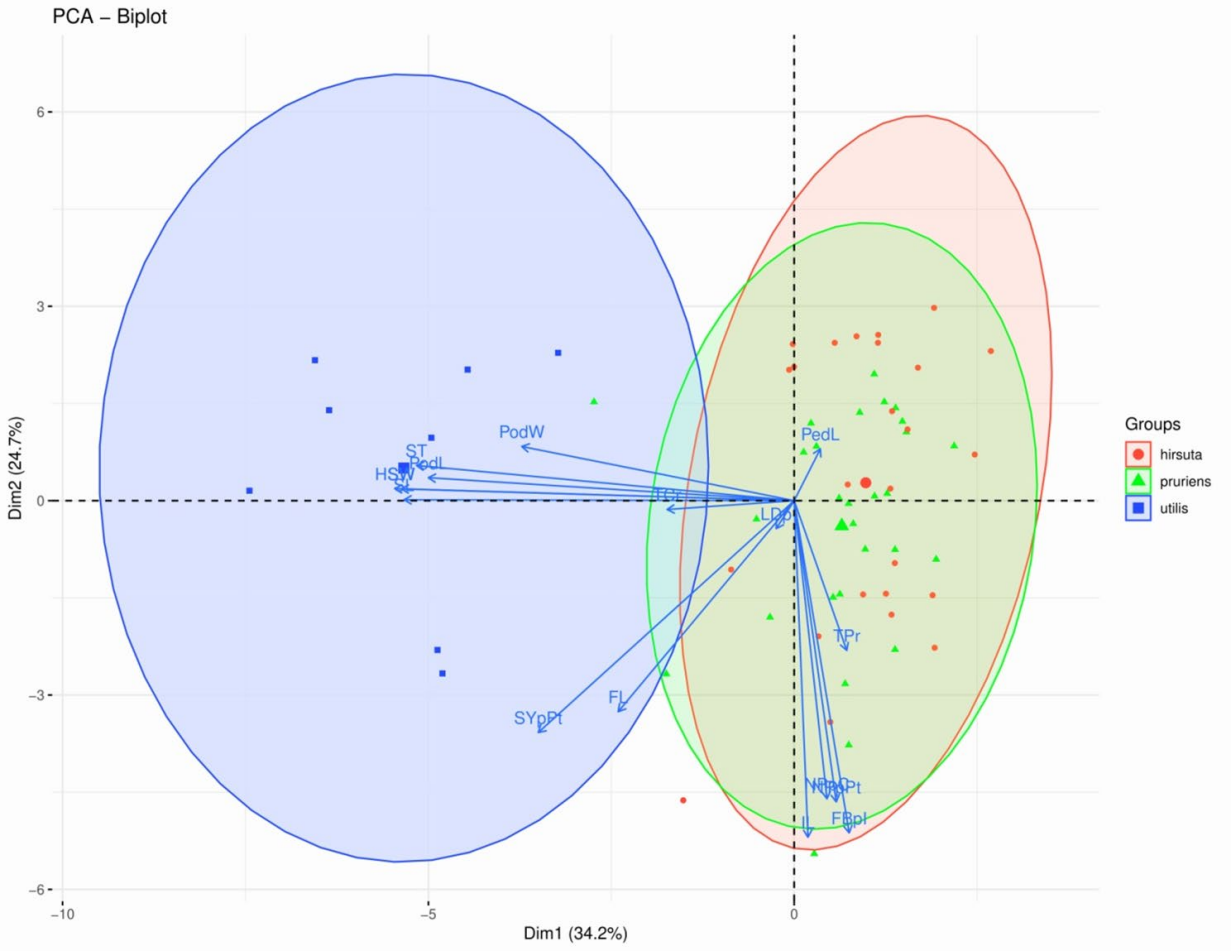
Scatter plot of *M. pruriens* accessions based on sixteen phenotypic traits from the principal component analysis (PCA).*Note*: Red circles, green triangles and blue squares represent *M. pruriens* var. *hirsuta*, var. *pruriens*, and var. *utilis*, respectively

In principal component analysis (PCA), the first five principal components (PCs) explained 81.39% of the total phenotypic variance, of which PC1 accounted for 34.15%, and PC2 accounted for 24.68% (Fig. 2) (Table S3). The resultant scatter plot distinctly separated the cultivated and wild varieties. However, we couldn’t find clear-cut separation for the two wild varieties - var. *pruriens* and var. *hirsuta* (Fig. 2). We observed moderate to high estimates (42.86–99.93%) of broad-sense heritability (H^2^) for the 16 phenotypic traits (Table 1). Traits such as the number of pods per cluster, number of pods per plant, hundred seed weight, seed yield per plant, etc., showed high heritability values ranging from 93.34 to 99.93%. Among the biochemical traits, total protein (98.58%) and total carbohydrate (95.71%) recorded high heritability, and L-DOPA content revealed a moderately high heritability (62.50%).

### Genetic diversity

Initially, 90 markers tested on 70 accessions of *M. pruriens* (Table S4) produced 298 alleles with an average of 3.39 alleles per locus. At this stage, we applied a stringent filtering criterion to retain only 180 SSR alleles contributed by 66 markers based on minor allele frequency > 0.05, along with 61 accessions, which showed maximum missing site < 20% for further analysis. The major allele frequency (MAF, revealed by filtered SSR data) varied from 0.50 to 0.94, with an average of 0.73 indicating high genetic diversity and polymorphism at the observed loci. The heterozygosity (Ho) values ranged from 0.00 to 0.59 with a mean value of 0.12, and the gene diversity (He) varied from 0.03 (MPU_122) to 0.73 (MPU_42) with a mean value of 0.36. The summary of the marker attributes at different loci and individual markers retained after stringent filtration are given in Table 2 and Table S5, respectively.

**Table 2:** Marker attributes of sixty-six SSR markers used in the study

Major allele frequency (MAF)	
Average	0.73
SSR marker with lowest MAF	0.5 (MPU_52)
SSR marker with highest MAF	0.94 (MPU_20, MPU_122)
**Observed heterozygosity (Ho)**	
Average	0.12
SSR marker with lowest Ho	0.00 (MPU_39, MPU_47, MPU_80)
SSR marker with highest Ho	0.59 (MPU_90)
**Expected heterozygosity (He)**	
Average	0.36
SSR marker with lowest He	0.03 (MPU_122)
SSR marker with highest He	0.73 (MPU_42)
**Polymorphism Information Content (PIC)**	
Average	0.29
SSR marker with the lowest PIC	0.09 (MPU_20)
SSR marker with the highest PIC	0.38 (MPU_52)

The polymorphism information content (PIC) value, which provides relative informativeness of each marker, ranged between 0.09 (for SSR marker MPU_22) and 0.38 (MPU_52) with an average of 0.29 (Table S5). The significant proportion of SSR markers in our study was moderately polymorphic (43 SSR markers with PIC-values ranging from 0.26 to 0.38, Table S5). The mean and private/rare allelic richness per marker were 2.82 and 0.06, respectively. The majority of the SSR markers deviated from the Hardy-Weinberg equilibrium (HWE) at a significance level of *P* < 0.001, and very few markers deviated at *P* < 0.01 (MPU_46 and MPU_115) and *P* < 0.05 (MPU_92) (Table S5). The SSR markers MPU_122 and MPU_130 showed no significant deviation.

Shannon’s information index (I = 0.62) and Nei’s gene diversity (h = 0.32) suggest high genetic diversity within our association mapping panel. The total gene diversity value (H_T_ = 0.34) (Table S5) substantiated this finding. For more perceptive analyses, we subdivided the association panel accessions into two groups: the first one based on three regional gene pools (Eastern, Central-West, and Southern India) and the second one on three botanical varieties (var. *utilis*, var. *pruriens*, and var. *hirsuta*). We independently estimated genetic diversity statistics for each gene pool (Table 3). In the case of regional gene pools, I varied from 0.56 to 0.67, and h ranged from 0.31 to 0.34 (Table 3). Accessions from West-Central India (I = 0.67, h = 0.34) and Southern India (I = 0.64, h = 0.34) were more diverse as compared to ones from Eastern India (I = 0.56, h = 0.31). The percentage of polymorphic SSR loci was 96.97% for the accessions of South India, followed by 89.39% and 87.88% for the accessions of West-Central and Eastern India, respectively. Among the three varietal gene pools, I varied from 0.44 to 0.72, and h ranged from 0.28 to 0.36 (Table 3). Wild varieties, var. *pruriens* (I = 0.72, h = 0.36) and var. *hirsuta* (I = 0.62, h = 0.34), were genetically more diverse than the cultivated var. *utilis* (I = 0.44, h = 0.28). The percentage of polymorphic SSR loci was 98.48%, 96.97%, and 72.73% in var. *pruriens*, var. *hirsuta*, and var. *utilis*.

**Table 3:** Genetic diversity estimates for the regional gene pools and botanical varieties within *M. pruriens* association mapping panel

Regional gene pool	*N*	Na	Ne	I	h	Ho	He	%*P*
Eastern India	15	2.48	1.65	0.56	0.31	0.09	0.33	87.88
West-Central India	11	2.56	1.95	0.67	0.34	0.13	0.40	89.39
Southern India	35	3.15	1.75	0.64	0.34	0.13	0.35	96.97
**Varietal gene pool**	**N**	**Na**	**Ne**	**I**	**h**	**Ho**	**He**	**%P**
*M. pruriens* var. *utilis*	8	1.98	1.53	0.44	0.28	0.06	0.27	72.73
*M. pruriens* var. *pruriens*	28	3.11	1.92	0.72	0.36	0.12	0.41	98.48
*M. pruriens* var. *hirsuta*	25	3.00	1.72	0.62	0.34	0.14	0.35	96.97

N: No. of accessions; Na: Total no. of alleles; Ne: Effective no. of alleles; I: Shannon’s information index; h: Nei’s gene diversity; Ho: observed heterozygosity; He: Expected heterozygosity; %P: Percentage of polymorphic loci

### Population structure

**Fig. 3:**
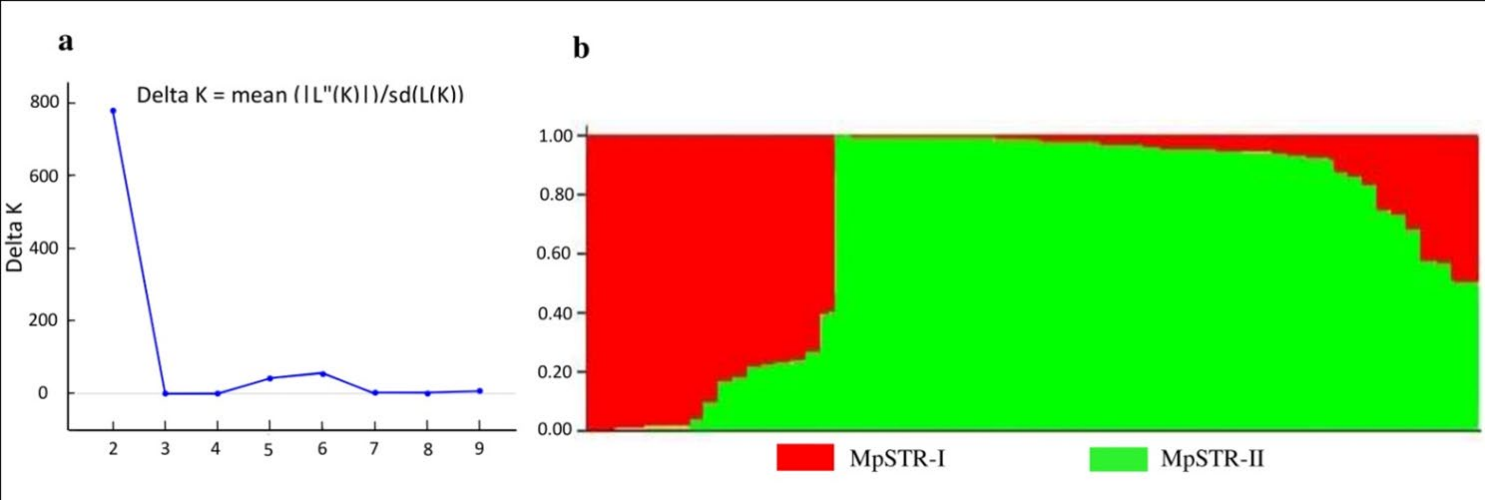
Population structure of *M. pruriens* association mapping panel inferred using STRUCTURE software **(a)** Hypothetical sub-population estimation using ∆K-values (K = 2) indicating two subpopulations **(b)** Population structure at K = 2 based on inferred ancestry (Q-matrix) in which two sub-populations are indicated as MpSTR-I (red color) and MpSTR-II (green color)

STRUCTURE analysis (∆K vs. K plot) revealed a sharp peak at K = 2, indicating the presence of two genetic groups (MpSTR-I and MpSTR-II) in our association mapping panel (Fig. 3a and b). Based on the geographical affiliation of accessions, subgroup-1 (designated as MpSTR-I) was composed of 17 accessions, of which 14 were derived from Eastern India, two from West-Central India, and one from Southern India. Similarly, subgroup-2 (designated as MpSTR-II) was composed of 44 accessions, of which 34 were derived from Southern India, nine from West-Central India, and one from Eastern India. This indicated that the majority of accessions belonging to Eastern India were present in MpSTR-I, and those belonging to Southern India were present in MpSTR-II. However, accessions from West-Central India did not form an independent subgroup but merged with one of the two major clusters. Based on the varietal affiliation, MpSTR-I was composed of five accessions of var. *utilis* and 12 accessions of var. *pruriens*. Likewise, MpSTR-II was composed of 25 accessions of var. *hairsuta*, 16 accessions of var. *pruriens*, and three accessions of var. *utilis*. Thus, independent grouping based on variety was also absent. Out of the total accession present in both of the subgroups, 48 accessions showed > 80% of shared ancestry, and 13 were admixtures with < 80% of shared ancestry (Fig. 3b).

To ascertain the partitioning of variation, we performed AMOVA on three population groups assumed based on (a) geographic distribution, (b) varietal affiliation, and (c) subgroups identified in STRUCTURE analysis (Table 4). All three revealed higher within gene pool variance than among gene pools. The ratios for within- to between-gene pool variance were 93:7 in the case of geographical distribution, 97:3 for varietal affiliations, and 89:11 for structure-derived sub-populations. We estimated population differentiation (F_ST_) within each group separately. The results revealed low to moderate differentiation in groups (pairwise F_ST_) based on the varietal affiliation (F_ST_ = 0.032) and geographic origin (F_ST_= 0.070) and high genetic differentiation (F_ST_= 0.105) in groups based on STRUCTURE analysis (Table 4).

**Table 4:** Population differentiation and AMOVA based on geographic distribution, varietal affiliation, and STRUCTURE analysis within *M. pruriens* association mapping panel

Geographic distribution					
Sources of variation	Estimated Variance	Percentage of Variance		*P*-value*	F_ST_
Among populations	1.048	7%		0.001	0.070
Within populations	13.841	93%		0.001	
Total	14.889	100%		0.001	
**Varietal affiliation**					
**Sources of variation**	**Estimated Variance**	**Percentage of Variance**		***P*** -value*	**F**_**ST**_
Among populations	0.463	3%		0.001	0.032
Within populations	14.172	97%		0.001	
Total	14.635	100%		0.001	
**STRUCTURE**					
**Sources of variation**	**Estimated Variance**	**Percentage of Variance**		***P*** -value*	**F**_**ST**_
Among populations	1.619	11%		0.001	0.105
Within populations	13.794	89%		0.001	
Total	15.413	100%		0.001	

*: With 999 data permutations

**Fig. 4:**
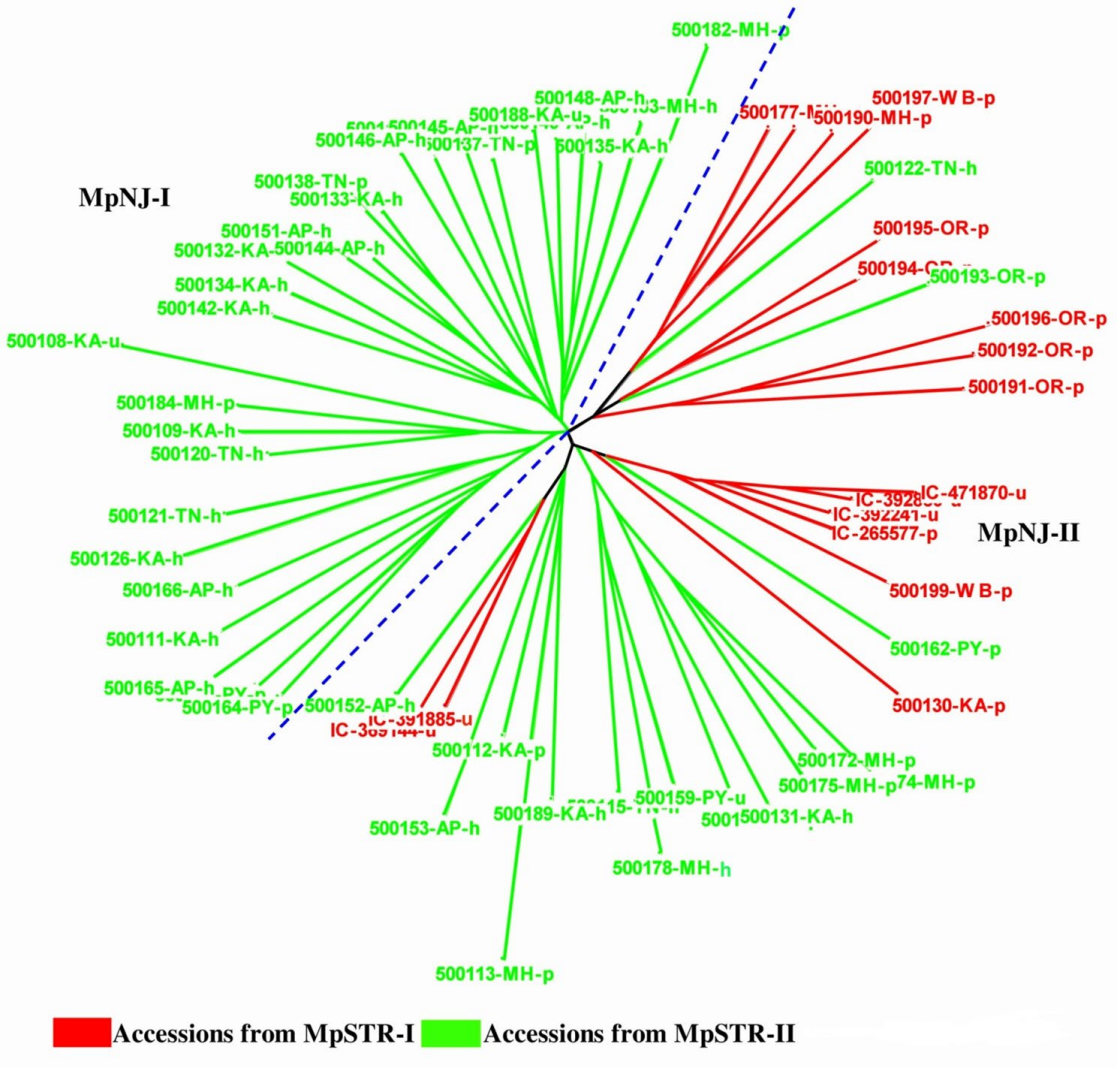
Neighbor joining (NJ) dendrogram based on the genetic distance. *Note*: Red and green color indicates subgroup-1 (MpSTR-I) and subgroup-2 (MpSTR-II) from STRUCTURE analysis

We constructed the NJ dendrogram using a simple matching coefficient to determine the genetic relationship and performed PCoA. The NJ algorithm revealed two main clusters, MpNJ-I and MpNJ-II (Fig. 4). Similar to STRUCTRE results, most of the 28 accessions in MpNJ-I were derived from Southern India (25 accessions). However, considerable mixing was observed among the 33 accessions in MpNJ-II, as 15 belonged to Eastern India, nine were from West-Central India, and nine were from Southern India. The accessions in the NJ tree and PCoA plots (PC1 vs. PC2) are color-coded from the information of two subgroups identified using the STRUCTURE analysis (MpSTR-I and MpSTR-II). We didn’t find any indication of grouping based on geographic origin or varietal affiliation, as accessions representing these groups were generally mixed.

**Fig. 5:**
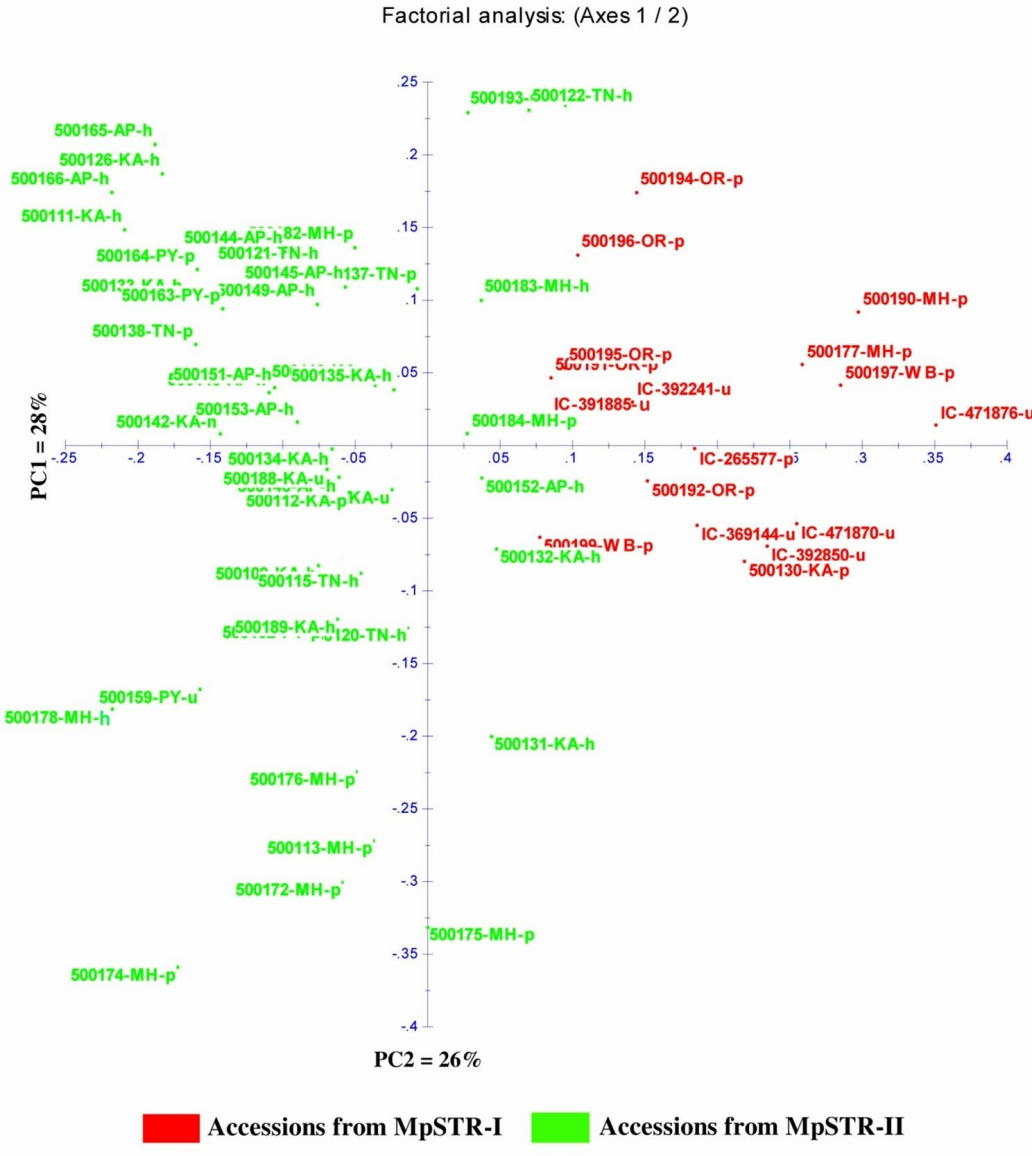
Scatter plot depicting dispersion of M. pruriens accessions based on principal coordinate analysis. *Note*: Red and green color indicates subgroup-1 (MpSTR-I) and subgroup-2 (MpSTR-II) from STRUCTURE analysis

**Fig. 6:**
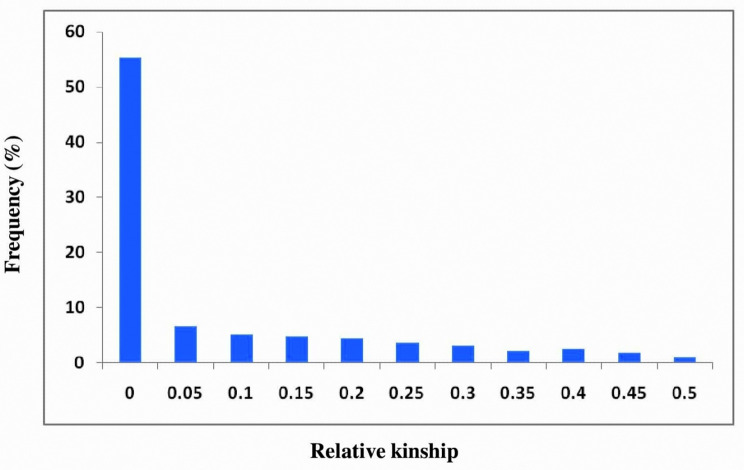
Distribution of global pairwise kinship coefficients (*F*_*ij*_*)* of *M. pruriens* association panel

**Fig. 7:**
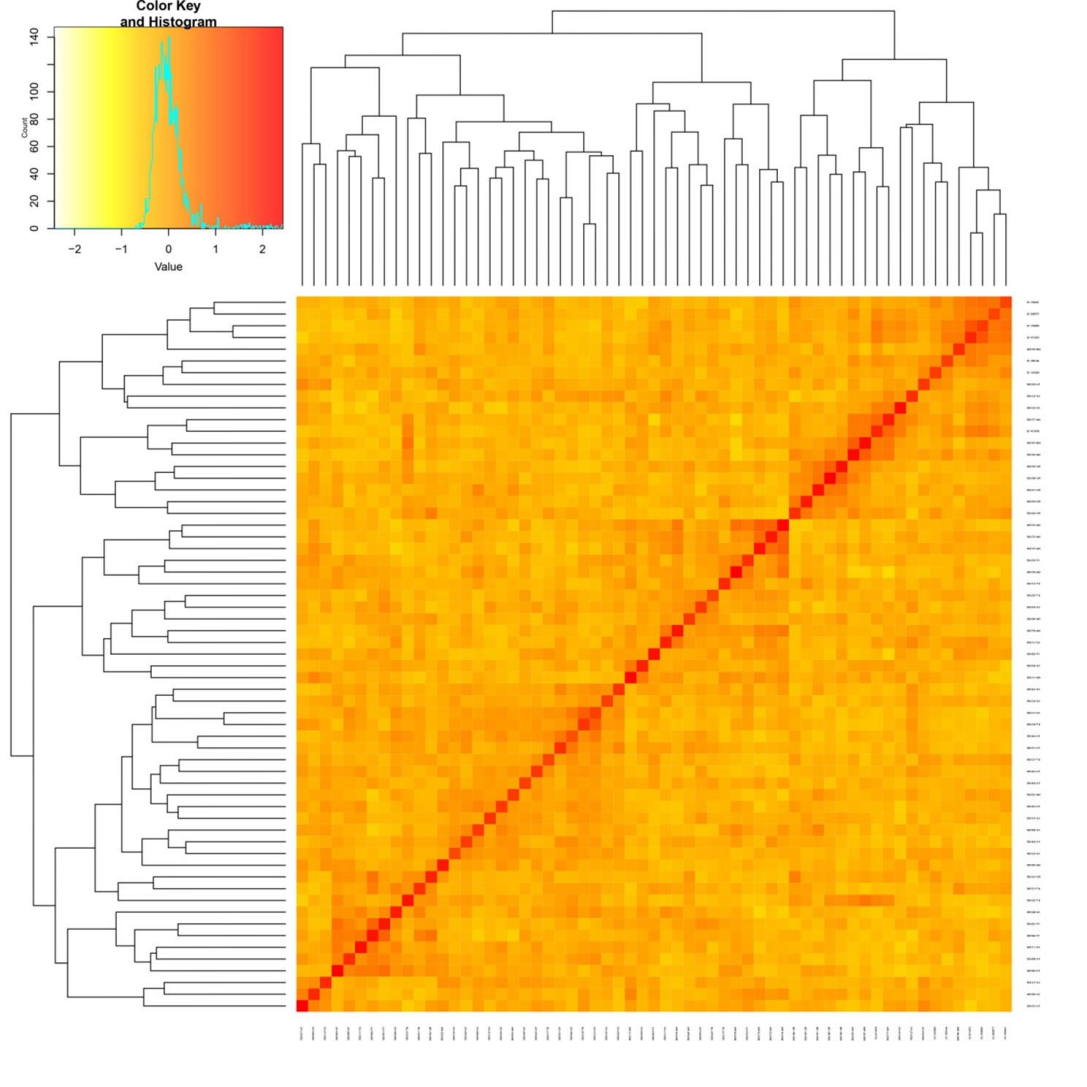
Heat map of kinship matrix generated for *M. pruriens* association mapping panel based on 180 filtered SSR markers data. Dendrogram are shown on the top and left side of the figure

In principal coordinate analysis (PCoA), the principal axes 1 and 2 explained 28 and 26% of the total variance (Fig. 5). The dispersion was relatively homogeneous in all four quadrants, signifying the diverse nature of the accessions. The clustering pattern in PCoA largely corresponded with that of the NJ-dendrogram. We also estimated the relative kinship between these accessions to measure their relatedness. About 55.29% of kinship coefficient (F_ij_) values between any two accessions were within 0 to 0.05, and there was a subsequent reduction in frequency with an increase in kinship value (Fig. 6). The heat map (Fig. 7) revealed substantial differences among the accessions.

### Association mapping

**Fig. 8:**
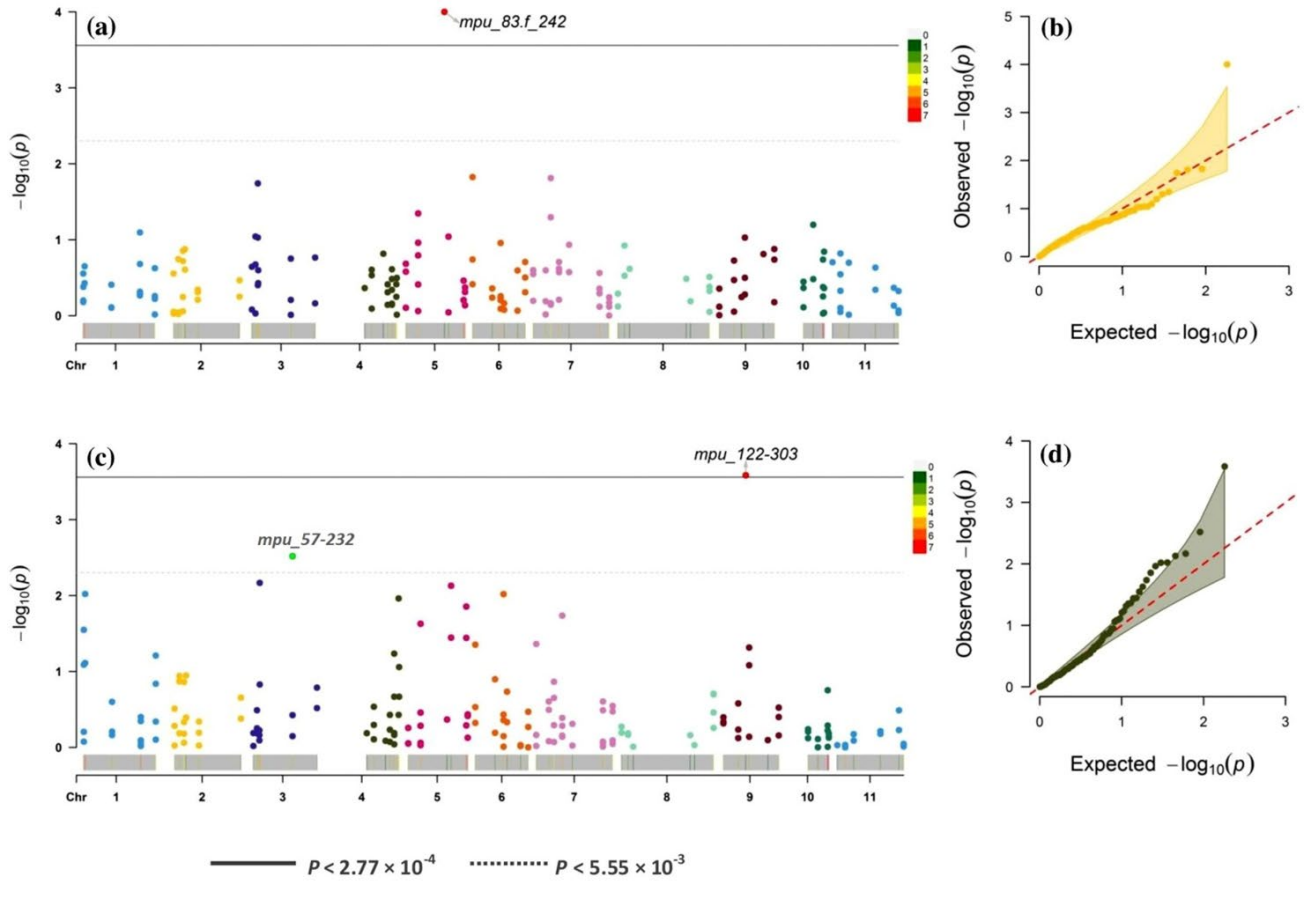
Manhattan plot (*P*-value) for MLM depicting significant marker-trait associations at adjusted Bonferroni threshold *P* < 2.77 × 10^− 4^**(a)** Seed yield per plant **(b)** Pedicel length; Quantile-Quantile (Q-Q) plot of MLM **(c)** Seed yield per plant **(d)** Pedicel length

For association analysis, we employed the mixed linear model (MLM), which integrates both population structure (Q matrix) and kinship (K matrix) to avoid false-positive MTAs. Only two MTAs involving two SSR markers (MPU_83 and MPU_122) passed the stringent Bonferroni adjusted threshold *P* < 2.77 × 10^− 4^ (*P* = 0.05/180, where 180 is the number of markers used in the analysis). These two associations were for the seed yield per plant (phenotypic variance explained, PVE = 31.12%) and the pedicel length (PVE = 25.07%) (Fig. 8 a-d).

We also used a less stringent criterion of *P* < 5.55 × 10^− 3^ (1/180) as the cut-off for the reasons explained in the method section. By applying, a total of 15 additional MTAs related to inflorescence length, flower buds per inflorescence, flower length, pedicel length, seed length, seed width, seed thickness, hundred seed weight, and seed yield per plant were identified (Table 5). The PVE for these MTAs ranged from 14.72 to 31.12%. These MTAs were contributed mainly by 10 SSR markers: MPU_19, MPU_42, MPU_54, MPU_57, MPU_58, MPU_83, MPU_89, MPU_108, MPU_111, and MPU_122. We found four MTAs contributing to a hundred seed weight, two each for seed length, seed width, pedicel length, and at least one each for inflorescence length, flower buds per inflorescence, flower length, seed thickness, and seed yield per plant. The highest PVE (31.12%) was observed in MTA between SSR marker MPU_83 and an important agronomic trait, seed yield per plant. This crucial MTA was also spotted at Bonferroni adjusted threshold *P* < 2.77 × 10^− 4^ (Fig. 8a). The Manhattan and Q-Q plots of MTAs at *P* < 5.55 × 10^− 3^ are given in Fig. S1 and S2, and the MTAs significant at threshold *P* < 0.05 and PVE > 10% are provided in Table S6.

**Table 5:** Marker-trait associations identified using mixed linear model (MLM) at *P* < 5.55 × 10^− 3^ with phenotypic variance explained (PVE) > 10%

Trait	Marker	*P*-value	PVE (%)
Inflorescence length	MPU_58	4.50 × 10^− 3^	14.72
Flower buds per inflorescence	MPU_19	2.33 × 10^− 3^	19.43
Flower length	MPU_19	4.09 × 10^− 3^	15.48
Pedicel length	MPU_57	3.03 × 10^− 3^	15.81
	MPU_122	2.60 × 10^− 4^	25.07
Seed length	MPU_42	4.35 × 10^− 3^	15.43
	MPU_83	4.99 × 10^− 3^	14.94
Seed width	MPU_42	5.20 × 10^− 3^	15.61
	MPU_83	2.55 × 10^− 3^	18.48
Seed thickness	MPU_54	5.04 × 10^− 3^	15.15
Seed yield per plant	MPU_83	1.00 × 10^− 4^	31.12
Hundred seed weight	MPU_54	2.88 × 10^− 4^	26.70
	MPU_89	1.10 × 10^− 3^	20.27
	MPU_108	7.68 × 10^− 4^	22.08
	MPU_111	2.78 × 10^− 3^	16.85

PVE: Phenotypic variance explained

The association analysis also revealed four SSR markers associated with multiple agronomic traits (*P* < 5.55 × 10^− 3^; PVE > 10%). Marker MPU_83 was not only associated with the seed length and seed width but also with the seed yield per plant. SSR loci MPU_54 was associated with seed thickness and hundred seed weight. MPU_42 was associated with seed length and seed width, and MPU_19 was associated with flower length and flower buds per inflorescence. These MTAs were justified by high significant correlations (*P* < 0.05) values between these traits of agronomic importance (Fig. 1).

### Annotation of marker-trait associations

To test the reliability of our MTAs, we determined the putative gene/function for each trait-associated marker (*P* < 5.55 × 10^− 3^; PVE > 10%) by annotating the related SSR primers using the *Arabidopsis thaliana* (L.) Heynh. genome database. Out of the ten trait-associated markers, the BLAST analysis annotated seven markers viz., MPU_19, MPU_42, MPU_54, MPU_89, MPU_108, MPU_111, and MPU_122 (Table 6). Notably, the SSR marker MPU_19, associated with flower length and flower buds per inflorescence, was annotated as 4-coumarate-CoA ligase 2, and MPU_42, associated with seed length and seed width, was annotated for Zinc transporter 7-precursors. Likewise, the SSR marker MPU_54 associated with traits such as seed thickness and hundred seed weight was annotated for sucrose non-fermenting 1- related protein kinase 2 (SnRK2) gene, and MPU_89 associated with hundred seed weight was annotated as 3-dehydroquinate synthase. The SSR MPU_108, associated with hundred seed weight, matched with NAC domain-containing protein, a plant-specific transcription factor (TF) associated with multiple aspects of stress and development. Further, MPU_111 (hundred seed weight) and MPU_122 (pedicel length) were annotated with Ubiquitin-specific protease and LEUNIG homolog (LUG and LUH), respectively.

**Table 6:** Putative gene ID/description of the trait-associated markers obtained from *Arabidopsis* annotated genes using blast results

Markers	*P*-value	Associated Traits	Gene ID/Description	Functions attributed
MPU_19	0.00671	FBpI, FL	4-coumarate-CoA ligase 2 *(4CL; EC 6.2.1.12)*	Catalyzes the activation of 4-coumarate and a few related substrates to the respective CoA esters and thus channels the common, phenylalanine-derived building block into the otherwise widely distinct branches of general phenylpropanoid metabolism (Hamberger and Hahlbrock 2004).
MPU_42	0.00326	SL, SW	Zinc transporter 7 precursor (*AT2G04032*)	Mediates zinc uptake from the rhizosphere (Grotz et al. 1998; Milner et al. 2013).
MPU_54	6.95E-04	ST, HSW	Sucrose non-fermenting 1- related protein kinase 2 (SnRK2)	Involved in the abscisic acid (ABA) signaling and plays a central role in plant stress signal transduction (Feng et al. 2018).
MPU_89	0.00294	HSW	3-dehydroquinate synthase	Catalyzes the transformation of the seven-carbon sugar 3-deoxy-D-*arabino*-heptulosonate 7-phosphate (DAH7P) into the carbocycle dehydroquinate (DHQ) (Negron et al. 2011).
MPU_108	0.00133	HSW	NAC domain-containing protein	Plant-specific transcription factors (TFs) are associated with multiple aspects of the stress and development region (Mathew et al. 2016).
MPU_111	0.00311	HSW	Ubiquitin-specific protease 23 *(AT5G57990)*	The organ size in plants is reported to be regulated by two reversible processes called ubiquitination and deubiquitinating (Shi et al. 2019).
MPU_122	1.53E-04	PedL	LEUNIG homolog (LUG and LUH)	The two proteins may act cooperatively to coordinate inflorescence architecture through their influences on auxin biosynthesis, transport, and perception (Douglas et al. 2017).

FBpI: Flower buds per inflorescence; FL: Flower length; SL: Seed length; SW: Seed width; ST: Seed thickness; HSW: Hundred seed weight; PL: Pedicel length

## Discussion

### Phenotypic diversity and heritability

*M. pruriens* is an underutilized legume species; hence, its germplasm is maintained by a few international and national research institutes/organizations such as the International Institute of Tropical Agriculture (IITA), Nigeria; United States Department of Agriculture (USDA), the USA; and the National Bureau of Plant Genetic Resources (NBPGR), India (Jorge et al. 2007). We used the most diverse and informative available germplasm collection, containing 70 accessions, as an association mapping panel. We found significant variability for most of the flower, pod, seed, and biochemical characteristics in our association mapping panel. This is consistent with earlier reports in different *M. pruriens* germplasm (Gurumoorthi et al. 2003; Kalidass and Mohan 2011; Sathyanarayana et al. 2012). The majority of the seed and pod-based traits revealed a significant positive correlation, possibly due to the critical role played by the pod in seed development in terms of protection and nutrient source (Bennett et al. 2011). Several accessions such as IC-369,144 (27.89%), 500,113-MH (28.05%), 500,126-KA (28.55%), 500,135-KA (28.76%), 500,178-MH (29.11%), 500,184-MH (29.19%), and 500,190-MH (29.12%) showed a seed protein content akin to soybean (35–40%; Michelfelder 2009). These stocks will be helpful in breeding for protein content. But, unlike previous studies (Tripathi et al. 2018; Kumar et al. 2019), we found less variability for the L-DOPA content in our germplasm. The estimation method used (Daxenbichler et al. 1972) in this study is old and laborious; thus, it is plausible that poor recovery and associated shortcomings might have skewed this experiment. Moderately high heritability (H^2^ > 60%) was observed for different traits in our study. Two earlier studies found analogous observations for traits such as pod length, pod width, pod weight, hundred seed weight, seed yield per plant, and inflorescence length (Hadapad et al. 2018; Chinapolaiah et al. 2019). Our results reinforce them. High heritability means less influence of the environmental factors rendering phenotypic selection reliable, besides contributing to high additive effect in the breeding programs (Tiwari et al. 2011; Rosmania et al. 2016).

### Genetic diversity, population structure, and kinship analysis

Initially, we used 90 SSR markers on 70 *M. pruriens* accessions, but after stringent filtration, only 66 SSR markers and 61 accessions were retained for further analysis. These markers showed a moderate PIC value (0.25 < PIC < 0.50). This corroborates with the earlier report in *M. pruriens* (Kumar et al. 2019), albeit with a slightly higher allelic range and allele per locus. The result endorses the choice of SSR markers for our study. The Shannon’s information index suggests high genetic diversity (I = 0.62). This is higher than the values reported using AFLP (0.34) and SSR (0.47) markers in earlier studies (Tripathi et al. 2018; Kumar et al. 2019). In a predominantly self-pollinating species like *M. pruriens*, we expect lower levels of genetic diversity. However, higher values recorded here and in some earlier studies (summarized in Sathyanarayana et al. 2016) categorically point to the presence of out-crossing in this species, as Padmesh et al. (2006) reported. This is partly supported by a high average gene flow among the population groups (Nm = 3.03). Thus, further studies on the pollination mechanism in *M. pruriens* can throw more light on the drivers of genetic diversity in this species. Among the three geographical regions, accessions from West-Central and Southern India revealed higher diversity than that of Eastern India, consistent with Kumar et al. (2019). Of the three botanical varieties, wild varieties (var. *pruriens* and var. *hirsuta*) were more diverse than the cultivated variety (var. *utilis*) – the finding often documented in many earlier studies (Leelambika and Sathyanarayana 2011; Sathyanarayana et al. 2012; Tripathi et al. 2018; Kumar et al. 2019). Nonetheless, wide phenotypic and genetic diversity in our germplasm collection signifies its utility for the association analysis.

The low to moderate F_ST_ values (0.032, 0.070) in *M. pruriens* varietal and geographical populations indicated less divergence. The population structure analyses from the distance-based NJ-tree, PCoA, and Bayesian-based STRUCTURE largely conformed to each other in suggesting the minimal influence of the geographical origin and/or the varietal affiliation on the grouping of the accessions. High gene flow estimates (Nm = 3.03), the presence of genetic admixtures, and higher within-population variance in AMOVA further concur with this. These observations are also chronicled in different germplasm collections in earlier studies (summarized in Sathyanarayana et al. 2016). Together, these studies and our results suggest one or more explanations: extensive pollen flow across long geographic distances, lack of inter-varietal barriers for hybridization, long seed dispersal, and high seed germination rates. The species thus appears to be highly adaptable, as evident from its broad and diverse distribution range. Alternatively, this might suggest incomplete lineage sorting during its diversification, as observed in safflower (*Carthamus tinctorius* L.) (Ambreen et al. 2018). However, we caution that our experiments are based on the limited sample size. Future studies must examine more samples from a broad geographical range to confirm these hypotheses. Further, we noticed a low pairwise kinship estimates, suggesting a weak relationship between most *M. pruriens* accessions in our association mapping panel. Low population divergence and kinship estimates further support the suitability of our panel for association analysis.

### Association mapping and significant marker-trait associations

Among the recent approaches used for fine-scale mapping of the desirable traits, association mapping has produced fast and reliable results in several legume taxa. This includes markers for seed-related traits in peanuts (*Arachis hypogaea* L.) (Zhao et al. 2017); iron and zinc concentration in lentils (*Lens culinaris* Medikus) (Singh et al. 2017); frost tolerance in peas (*Pisum sativum* L.*)* (Liu et al. 2017); flowering, pod yield per plant and fresh pod per pant in lablab [*Lablab purpureus* (L.) Sweet] (Vaijayanthi et al. 2018); crude protein and mineral concentration in alfalfa (*Medicago sativa* L.) (Jia et al. 2017); etc. However, in *M. pruriens*, association mapping has never been used (Lepcha et al. 2019), perhaps due to a lack of resources. Thus, we attempted association mapping of key agronomic traits in *M. pruriens* in the present study. Usually, a large number of accessions (> 300) are used in association studies. In comparison, our number is small (61). However, several recent studies have demonstrated the feasibility of finding the right marker-trait associations even using a small number of accessions (Lehnert et al. 2018; Ma et al. 2018; Rohilla et al. 2020; Soumya et al. 2021).

We identified 15 significant MTAs (*P* < 5.55 × 10^− 3^) using MLM, with PVE ranging from 14.7%to 31.1%. These associations also benefitted by the moderate to high heritability observed for all the trait-associated markers (H^2^ ranging from 42.86 to 97.27%). Four SSR markers were associated with multiple traits. They are mainly comprised of seed or inflorescence traits that are highly correlated. Such an account of a single marker associated with various traits can be attributed to closely linked QTLs affecting different traits (Rakshit et al. 2010). Alternatively, it may also be due to the pleiotropic effect of the linked QTLs on other traits (Miller and Rawlings 1967).

### Annotation of SSR markers associated with the traits

We investigated the putative functions of the trait-associated markers by annotating against corresponding genes/loci in the *Arabidopsis thaliana* (L.) Heynh. database (https://www.arabidopsis.org). The results produced some useful insights. For instance, the SSR marker MPU_111, associated with a hundred seed weight, corresponded with the ubiquitin-specific protease 23 gene in *A. thaliana*. Another gene belonging to the same family in rice (*Oryza sativa* L.), ubiquitin-specific protease 23, is known to exert a positive regulatory influence on grain width and size (Shi et al. 2019). SSR marker MPU_54 associated with traits such as seed thickness and hundred seed weight was annotated for sucrose non-fermenting 1-related protein kinase 2 (SnRK2) gene. This gene is involved in the abscisic acid (ABA) signaling during seed germination, dormancy, and seedling growth and has a central role in plant stress signal transduction (Feng et al. 2018). The SSR MPU_108, associated with a hundred seed weight, was traced to NAC domain-containing protein, a plant-specific transcription factor (TF) associated with the multiple aspects of stress and development. It is reported that three NAC TF encoding genes (*ONAC020, ONAC026*, and *ONAC023)* are expressed at very high levels during the seed development in rice and exhibit a strong association with the seed size or weight with the sequence variations located in the upstream regulatory region (Mathew et al. 2016). SSR marker-MPU_122 associated with pedicel length was annotated for LEUNIG homolog (LUG and LUH). Likely, these two proteins act cooperatively to coordinate inflorescence architecture through their influence on auxin biosynthesis, transport, and perception (Douglas et al. 2017). The marker MPU_89 associated with hundred seed weight was annotated to the 3-dehydroquinate synthase gene, which catalyzes the transformation of the seven-carbon sugar 3-deoxy-D-arabino-heptulosonate 7-phosphate (DAH7P) into the 3-dehydroquinate (DHQ) (Negron et al. 2011). This pathway controls the production of precursors of aromatic amino acids in several prokaryotes, fungi, and plants (Ganem 1978). Scrutinizing its relationship with L-DOPA biosynthesis in *M. pruriens* might open fascinating insights into the regulation of aromatic amino acids, particularly tyrosine production during L-DOPA production. Thus, MTAs identified in the present study are candidates for experimental studies and upon validation will be useful for improving the seed yield and related economic traits in *M. pruriens*. To our understanding, this is the first report on marker-trait association based on association mapping in any *Mucuna* species from anywhere in the world.

However, our study has the following limitations: (a) Firstly, we have used a smaller number of accessions and SSR markers as the species has limited germplasm and genomic resources, and (b) Secondly, we have used single environment data for our analysis. So, there is a need to analyze the stability and utility of these marker-trait associations with more accessions and markers under additional environments through multi-location trials. Future studies must also focus on developing MTAs for other vital traits relevant to this crop, particularly indeterminate growth habit - a significant detriment in commercial cultivation of this crop and deduce the location of these MTAs on different linkage groups using bi-parental populations.

## Conclusion

In summary, a high phenotypic/genotypic diversity, low population divergence, moderate to high heritability of the evaluated traits, and low kinship estimates in our association mapping panel confirm its utility for association mapping. Fifteen MTAs identified for important traits with PVE > 10% from MLM offer a solid resource base for improving this crop. Their reliability tested through annotation against the *Arabidopsis* genome database lends further credence to this. Thus, the results from this study offer significant groundwork for future marker-assisted breeding and mining candidate genes for important agronomic traits in *M. pruriens*, a promising underutilized legume for the tropics.

### Electronic supplementary material

Below is the link to the electronic supplementary material.

Supplementary Material 1

Supplementary Material 2

## Data Availability

The datasets generated during and/or analyzed during the current study are available in the supplementary information section.
