# Supplementary material for: Association mapping of important agronomic traits in Mucuna pruriens (L.) DC
Source: Bot Stud. 2024 Aug 19;65:26. doi: 10.1186/s40529-024-00421-3 (PMC11333416; doi:10.1186/s40529-024-00421-3)
Supplement: Supplementary file 1 — Supplementary Material 1 [file 40529_2024_421_MOESM1_ESM.docx]

**Botanical Studies**

**Association mapping of important agronomic traits in *Mucuna pruriens* (L.) DC.**

**Supplementary Information**

All Supplementary Tables are combined in a single Excel file.

The table titles and captions are listed below.

**Table S1** Details of *M. pruriens* accessions used for the association analysis along with collection locations and geographic affiliations before filtration.

**Table S2** Details of SSR primer pairs used in the study before filtration.

**Table S3** Eigen vector and Eigen values of the first five principal components (PCs) for phenotypic traits.

**Table S4** Allelic data for 90 SSR markers.

**Table S5** Details of marker attributes based on 66 SSR markers retained after stringent filtration.

**Table S6** Marker-trait associations identified at a significance level of *P* < 0.05 with phenotypic variance explained (PVE) > 10%.

**
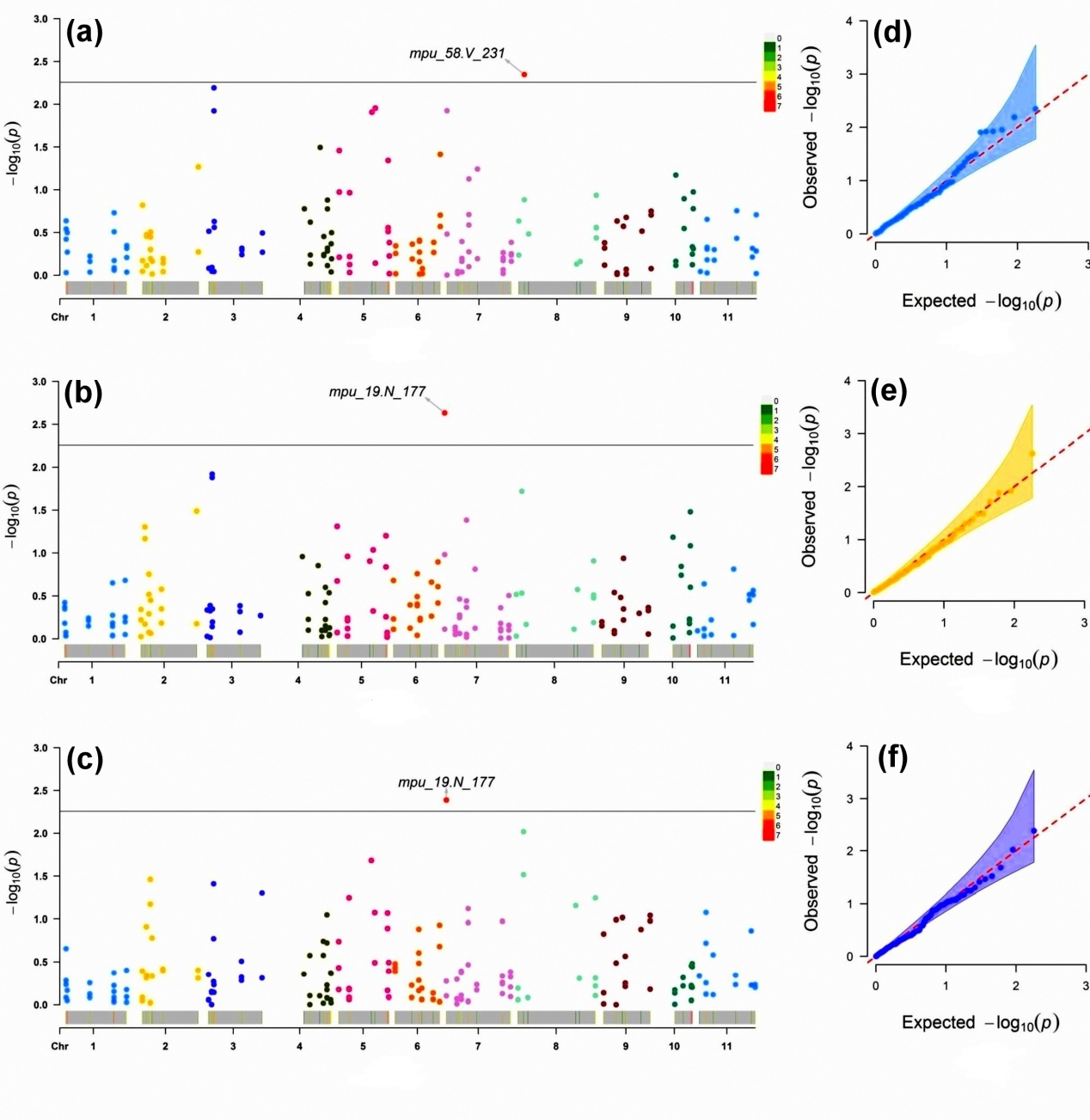
**

**Fig. S1** Manhattan plots (*P*-value) for MLM depicting significant marker trait association at adjusted threshold *P* < 5.55 × 10^-3^ **(a)** Inflorescence length **(b)** Flower buds per inflorescence **(c)** Flower length; Quantile-Quantile (Q-Q) plots of MLM **(d)** Inflorescence length **(e)** Flower buds per inflorescence **(f)** Flower length

**
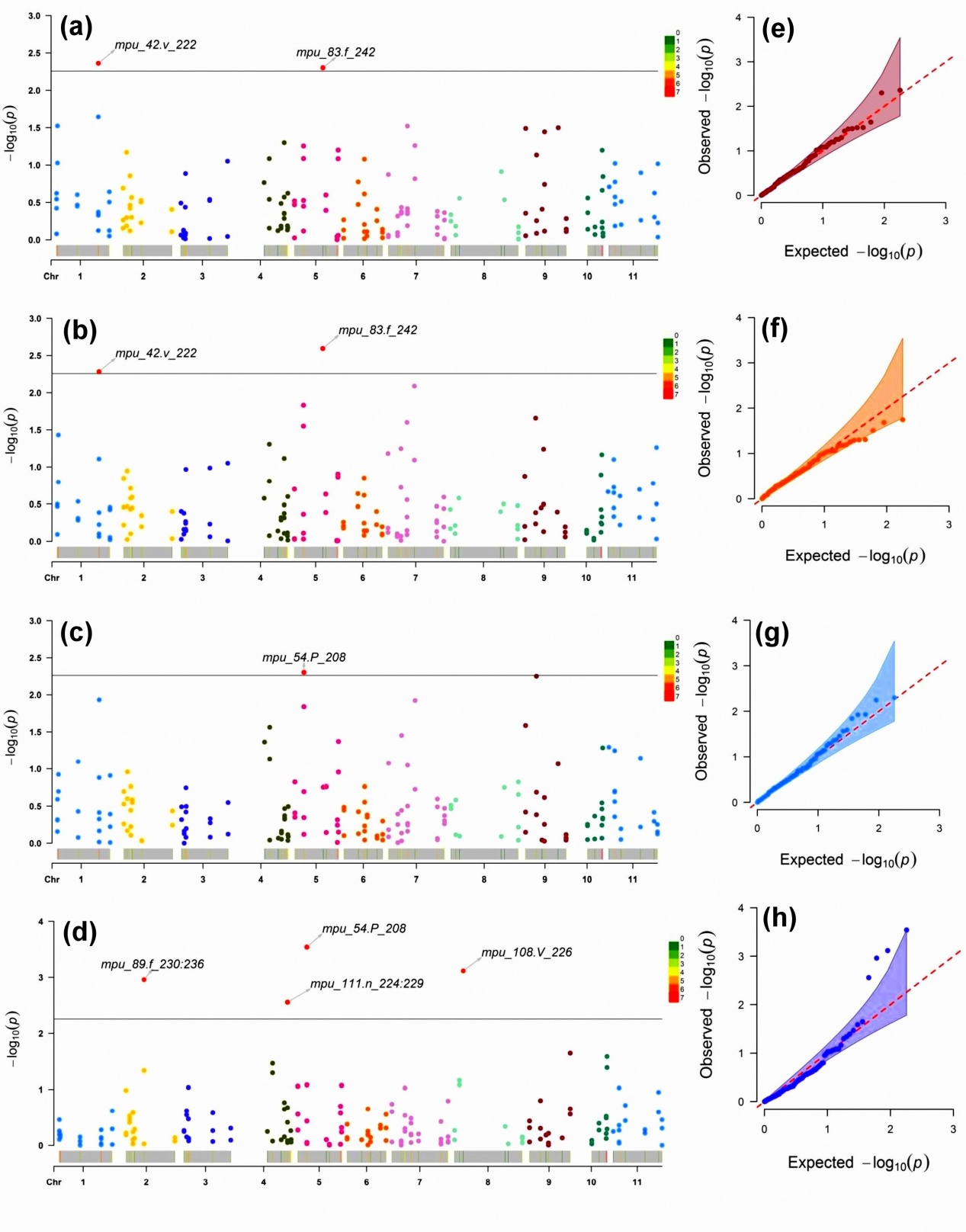
**

**Fig. S2** Manhattan plots (*P*-value) for MLM depicting significant marker-trait association at adjusted threshold *P* < 5.55 × 10^-3^ **(a)** Seed length **(b)** Seed width **(c)** Seed thickness **(d)** Hundred seed weight; Quantile-Quantile (Q-Q) plots of MLM **(e)** Seed length **(f)** Seed width **(g)** Seed thickness **(h)** hundred seed weight
